# Adipsin and adipocyte-derived C3aR1 regulate thermogenic fat in a sex-dependent fashion

**DOI:** 10.1172/jci.insight.178925

**Published:** 2024-05-07

**Authors:** Lunkun Ma, Ankit Gilani, Alfonso Rubio-Navarro, Eric Cortada, Ang Li, Shannon M. Reilly, Liling Tang, James C. Lo

**Affiliations:** 1 Division of Cardiology, Department of Medicine;; 2 Weill Center for Metabolic Health; and; 3 Cardiovascular Research Institute, Weill Cornell Medicine, New York, New York, USA.; 4 Key Laboratory of Biorheological Science and Technology, Ministry of Education, College of Bioengineering, Chongqing University, Chongqing, China.

**Keywords:** Inflammation, Metabolism, Adipose tissue, Complement

## Abstract

Thermogenesis in beige/brown adipose tissues can be leveraged to combat metabolic disorders such as type 2 diabetes and obesity. The complement system plays pleiotropic roles in metabolic homeostasis and organismal energy balance with canonical effects on immune cells and noncanonical effects on nonimmune cells. The adipsin/C3a/C3a receptor 1 (C3aR1) pathway stimulates insulin secretion and sustains pancreatic β cell mass. However, its role in adipose thermogenesis has not been defined. Here, we show that male *Adipsin/Cfd*-knockout mice exhibited increased energy expenditure and white adipose tissue (WAT) browning. In addition, male adipocyte-specific *C3aR1*-knockout mice exhibited enhanced WAT thermogenesis and increased respiration. In stark contrast, female adipocyte-specific *C3aR1*-knockout mice displayed decreased brown fat thermogenesis and were cold intolerant. Female mice expressed lower levels of *Adipsin* in thermogenic adipocytes and adipose tissues than males. *C3aR1* was also lower in female subcutaneous adipose tissue than in males. Collectively, these results reveal sexual dimorphism in the adipsin/C3a/C3aR1 axis in regulating adipose thermogenesis and defense against cold stress. Our findings establish a potentially new role of the alternative complement pathway in adaptive thermogenesis and highlight sex-specific considerations in potential therapeutic targets for metabolic diseases.

## Abstract 2

The alternative complement pathway regulates adipocyte metabolism via adipsin and C3aR1 in a sex-dependent manner.

## Graphical abstract

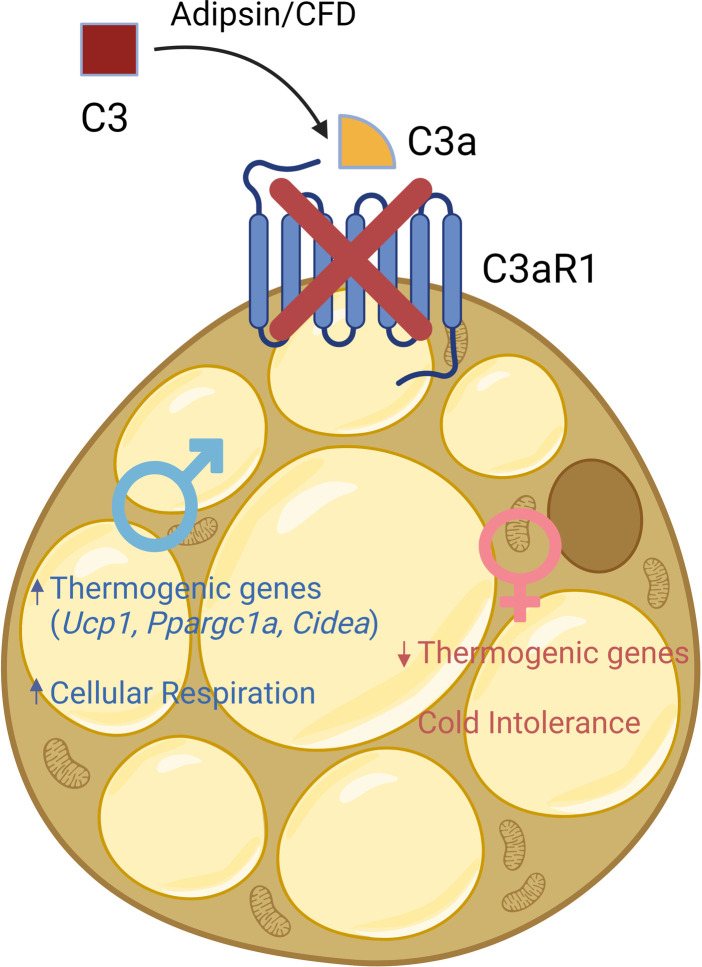

## Introduction

Obesity remains a serious global public health problem that greatly increases the risk of type 2 diabetes and other associated diseases, including cardiovascular disease and many types of cancer (1, 2). Bariatric surgery, currently the most effective durable option for treating severe obesity, carries risk of surgical complications, death, and reoperation (3). Glucagon-like peptide 1 receptor and gastric inhibitory polypeptide 1 receptor dual agonists are the most promising medical treatments for obesity to date, largely dependent on suppressing food intake (4, 5). Adaptive thermogenesis, the production of heat by the body in response to stimuli such as cold, is considered a promising therapeutic approach to counteract obesity (6). Thermogenic adipocytes in the brown and white adipose tissue depots are specialized cells that are responsible for adaptive thermogenesis by uncoupling protein 1 (UCP1) enhancing the leakage of protons across the mitochondrial membrane and other futile cycling pathways (7–9). Beige adipocytes embedded in white adipose tissues have multilocular lipid droplets and can promote thermogenesis like brown adipocytes (9, 10). Beige adipocytes can arise from both preadipocyte progenitors and preexisting mature unilocular white adipocytes (9, 11–15). The formation of beige adipocytes is activated by cold exposure, β-adrenergic stimulation, and other stimuli (9). The detection of brown adipose tissue (BAT) in human adults has further piqued interest in enhancing energy expenditure by increasing the thermogenic activity of brown and beige adipocytes (16–18). However, therapies to stimulate the thermogenic capacity of adipose tissues based on cold exposure or β-adrenergic agonists remain clinically challenging. Therefore, there is considerable interest in finding new mechanisms that promote browning and enhance thermogenesis in adipose.

The complement system consists of many distinct complement components that interact with one another. As an important part of the innate immune system, complement plays a key role in the defense against common pathogens (19). A growing body of research suggests that certain components of the complement system also play an important role in the regulation of metabolic disorders such as diabetes and insulin resistance (20–22). A critical complement component, complement factor D, also known as adipsin, is mainly secreted by adipocytes. Adipocytes synthesize the major components of the alternative complement pathway, but the role complement plays in adipose tissue homeostasis is unknown. Adipsin controls the alternative complement pathway by catalyzing the production of the C3 convertase, which then cleaves C3 to generate C3a and C3b (23). We previously showed that C3a acts to increase β cell insulin secretion, an effect that is dependent on C3a receptor 1 (C3aR1) (21, 24). Several studies show marked though opposing roles of adipsin and C3aR1 on systemic glucose homeostasis in diet-induced obesity (20, 21, 25). These results suggest that there may be different cell-specific effects of adipsin/C3aR1 on obesity and diabetes. C3aR1 is predominantly expressed on immune cells and was not previously known to be expressed by adipocytes. Furthermore, it is unknown whether the adipsin/C3a/C3aR1 axis is involved in the regulation of systemic energy homeostasis and adipose thermogenesis.

In the present study, we show that *Adipsin/Cfd* deficiency increased the thermogenic program in white adipose tissue (WAT) under ambient conditions and with cold exposure. We found that adipocytes expressed the anaphylatoxin receptor C3aR1. We generated adipocyte-specific *C3aR1*-knockout (Ad-*C3aR1^–/–^*) mice to elucidate the physiological roles of C3aR1 in adipocytes. Our study demonstrated that Ad-*C3aR1^–/–^* male mice exhibited enhanced thermogenic gene expression in subcutaneous adipose tissue. Male Ad-*C3aR1^–/–^* mice also showed increased heat production in thermogenic adipose tissue. Further in vitro studies showed that deletion of *C3aR1* in primary subcutaneous adipocytes from male mice enhanced thermogenic gene expression and mitochondrial respiration. Notably, we found sexual dimorphism in *Adipsin* and *C3aR1* gene expression. *Adipsin* was lower in both adipose tissue and thermogenic adipocytes of female mice compared with males. Subcutaneous adipose tissue *C3aR1* gene expression was also diminished in female mice compared with males. Furthermore, adipocyte-specific *C3aR1*-knockout (*C3aR1*-KO) female mice produced less heat in their BAT and were cold intolerant compared with controls. Our results reveal the function of the adipsin/C3a/C3aR1 axis as a regulator of adipose thermogenesis and energy metabolism in a sex-dependent manner.

## Results

### Loss of adipsin leads to increased energy expenditure and protection from diet-induced obesity.

**Figure 1:**
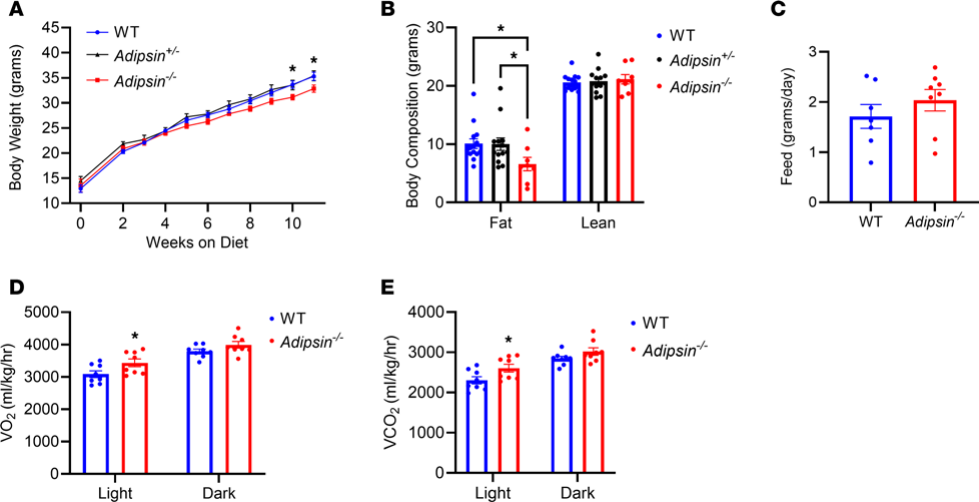
Mice deficient in Adipsin are protected from diet-induced obesity and display evidence of enhanced energy expenditure. (**A**) Body weights of wild-type (WT) and *Adipsin* heterozygous and *Adipsin*-knockout (KO) male mice on high-fat diet (HFD) for the indicated number of weeks. *n* = 8–14/group. Unpaired Welch’s *t* test is used for comparison between WT and KO groups. (**B**) Body composition of male mice from **A** after 12 weeks of HFD. *n* = 8–14/group. Two-way ANOVA with Tukey’s multiple comparisons is used for comparison between WT, heterozygous, and KO groups. (**C**) Food intake of male mice from **A** was measured daily after 4 weeks on HFD. *n* = 7–8/group. (**D** and **E**) O_2_ consumption (**D**) and CO_2_ production (**E**) rates of WT and *Adipsin*-KO male mice were measured by indirect calorimetry after 4 weeks on HFD. *n* = 7–8/group. Unpaired 2-tailed *t* test is used for comparison. Data are presented as mean ± SEM. *P < 0.05.

We previously found that adipsin positively regulates adipose tissue inflammation in mice fed a high-fat diet (HFD) (21). However, adipsin-deficient mice were mildly resistant to diet-induced obesity. To explore the potential role of adipsin in regulating whole-body energy homeostasis with metabolic stress, we compared the body weights of male wild-type (WT) and *Adipsin^–/–^* mice fed a regular diet or HFD. As expected, *Adipsin* mRNA levels were noticeably absent in all the fat tissues of the KO mice (Supplemental Figure 1A; supplemental material available online with this article; https://doi.org/10.1172/jci.insight.178925DS1). Although no change was seen in body weights between male WT and *Adipsin*^–/–^ mice (Supplemental Figure 1B) on a regular diet, *Adipsin*^–/–^ mice displayed a mild-moderate but significant reduction in body weight starting 2 months after HFD compared with both WT and *Adipsin*^+/–^ mice (Figure 1A). Body composition analysis showed that the reduced weight gain of *Adipsin*^–/–^ mice was mainly due to decreased fat mass without significant differences between WT and *Adipsin*^+/–^ mice (Figure 1B). As body weight is determined by a balance between energy expenditure and energy intake, we assessed food intake and energy expenditure in *Adipsin*^–/–^ and control mice at 4 weeks of HFD, before changes in body weight. Our results indicate that food intake was comparable between male WT and *Adipsin^–/–^* mice (Figure 1C), suggesting that the reduced body weight gain in *Adipsin^–/–^* mice was caused by increased energy expenditure. Indeed, *Adipsin^–/–^* mice exhibited an increase in O_2_ consumption and CO_2_ production compared with WT mice without significant differences in activity (Figure 1, D and E, and Supplemental Figure 1, C–E).

### Adipsin deficiency results in enhanced thermogenesis in white fat.

**Figure 2:**
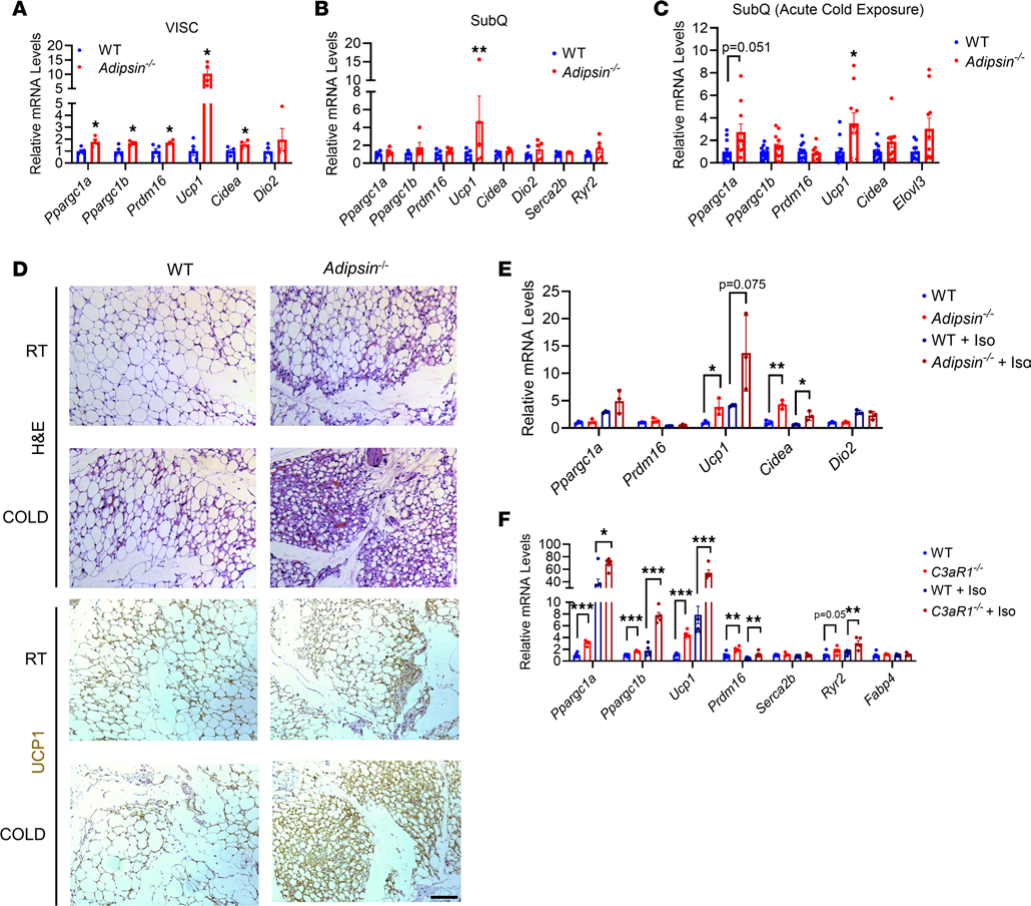
Adipsin deficiency promotes white adipose beiging. (**A** and **B**) Thermogenic gene expression in visceral (VISC) (**A**) and subcutaneous (SubQ) (**B**) fat of 10- to 12-week-old WT and *Adipsin*-KO male mice fed a regular diet at ambient temperature. *n* = 4–5/group. *Cidea*, cell death inducing DFFA like effector a; *Dio2*, deiodinase, iodothyronine, type II; *Ryr2*, ryanodine receptor 2, cardiac; *Elovl3*, ELOVL fatty acid elongase 3; *Fabp4*, fatty acid binding protein 4, adipocyte. (**C**) Thermogenic gene expression in SubQ fat of 10- to 12-week-old WT and *Adipsin*-KO male mice following an acute (6 hours) cold exposure. *n* = 10–11/group. (**D**) Hematoxylin and eosin (H&E) and UCP1 immunohistochemistry staining of inguinal white adipose tissue (WAT) sections from 10-week-old WT and *Adipsin*-KO male mice at room temperature (RT) and following acute cold exposure. Images are shown at 20× original magnification. Scale bar, 200 μm. (**E**) Thermogenic gene expression in primary SubQ adipocytes treated with or without isoproterenol (Iso) from WT and *Adipsin*-KO mice. *n* = 3/group. (**F**) Thermogenic gene expression in primary SubQ adipocytes treated with or without Iso from WT and *C3aR1*-KO mice. *n* = 5–6/group. Data are presented as mean ± SEM. Unpaired 2-tailed *t* test is used for comparison between WT and KO and between WT + Iso and KO + Iso groups for **E** and **F**. *P < 0.05, **P < 0.01, ***P < 0.001.

Because brown/beige adipocyte thermogenesis plays a key role in driving energy expenditure and adipsin is produced by adipocytes, we next examined whether there was enhanced adipose thermogenesis in *Adipsin^–/–^* mice. Ablation of adipsin resulted in increased expression of key thermogenic genes *Ucp1*, PPARγ coactivator 1α (*Ppargc1a*), *Ppargc1b*, and PR domain containing 16 (*Prdm16*) at room temperature in visceral (VISC) white adipose tissue (WAT) (Figure 2A), along with elevated *Ucp1* in subcutaneous (SubQ) WAT (Figure 2B). There was not a significant difference in thermogenic gene expression in the BAT between WT and *Adipsin^–/–^* mice (Supplemental Figure 2A).

SubQ WAT is prone to browning upon cold stimulation or β-adrenergic agonists (26). To further study the role of adipsin in regulation of white fat browning, we examined the effects of cold exposure on *Adipsin^–/–^* and WT mice. *Adipsin^–/–^* mice were similarly cold tolerant as controls (Supplemental Figure 2B). *Ucp1* was induced by 3-fold in the SubQ and VISC WAT of *Adipsin^–/–^* compared with WT mice following acute cold exposure (Figure 2C and Supplemental Figure 2C). However, the expression of thermogenic genes was not altered in the BAT of acute cold–exposed *Adipsin^–/–^* mice (Supplemental Figure 2D). Because of growing evidence that beige fat also uses alternative thermogenic pathways to dissipate energy in the form of heat (9), we next examined changes in *Ucp1*-independent thermogenic genes upon cold stimulation of SubQ fat. The *Adipsin^–/–^* group showed mild-moderately elevated expression of *Ldhb* and *Pkm* compared with controls (Supplemental Figure 2E). Consistent with the gene expression data, SubQ WAT from *Adipsin^–/–^* compared with that from WT mice showed more UCP1^+^ cells and reduced lipid stores (Figure 2D). After 1 week of chronic cold exposure, we did not detect differences in thermogenic gene expression between WT and *Adipsin^–/–^* mice (Supplemental Figure 2, F–H).

Because adipsin and its products can potentially act on many cell types, we next examined whether the increased numbers of thermogenic adipocytes in SubQ adipose tissue of *Adipsin*^–/–^ mice were cell autonomous. Adipocytes differentiated in vitro from the SubQ adipose depot from *Adipsin*^–/–^ mice also displayed a 4- to 5-fold increase in the expression of *Ucp1* and cell death, inducing *Cidea*, compared with controls (Figure 2E), suggesting an adipocyte cell–autonomous role for adipsin in thermogenesis. Furthermore, this increased expression of thermogenic genes in *Adipsin*^–/–^ cells was also enhanced by the β-agonist isoproterenol (Figure 2E).

Adipsin catalyzes the formation of the C3 convertase, which can then result in the cleavage of C3 into C3a and C3b (21, 23). C3a acts on cells through its G protein–coupled receptor C3aR1, whereas C3b activates the C5 convertase that can result in formation of the C5b-C9 membrane attack complex (21, 27–29). To determine whether adipsin regulates SubQ adipocyte browning acutely through its downstream C3a/C3aR1 signaling pathway, we next assessed primary SubQ adipocytes from WT and *C3aR1* whole-body KO mice. We found that SubQ adipocytes from *C3aR1*-KO mice differentiated in vitro showed a robust increase in the expression of thermogenic genes compared with WT controls (Figure 2F). Stimulating these cells with isoproterenol further augmented thermogenic gene expression in both groups, with *C3aR1*-KO adipocytes remaining 3- to 5-fold higher than controls (Figure 2F). These data suggest that adipsin through C3a/C3aR1 signaling regulates a browning phenotype in SubQ adipocytes. However, we did not observe baseline differences in O_2_ consumption and CO_2_ production in *C3aR1^–/–^* compared with WT mice (Supplemental Figure 2, I and J).

### Adipocyte C3aR1 regulates SubQ adipocyte thermogenic gene expression and mitochondrial respiration.

**Figure 3:**
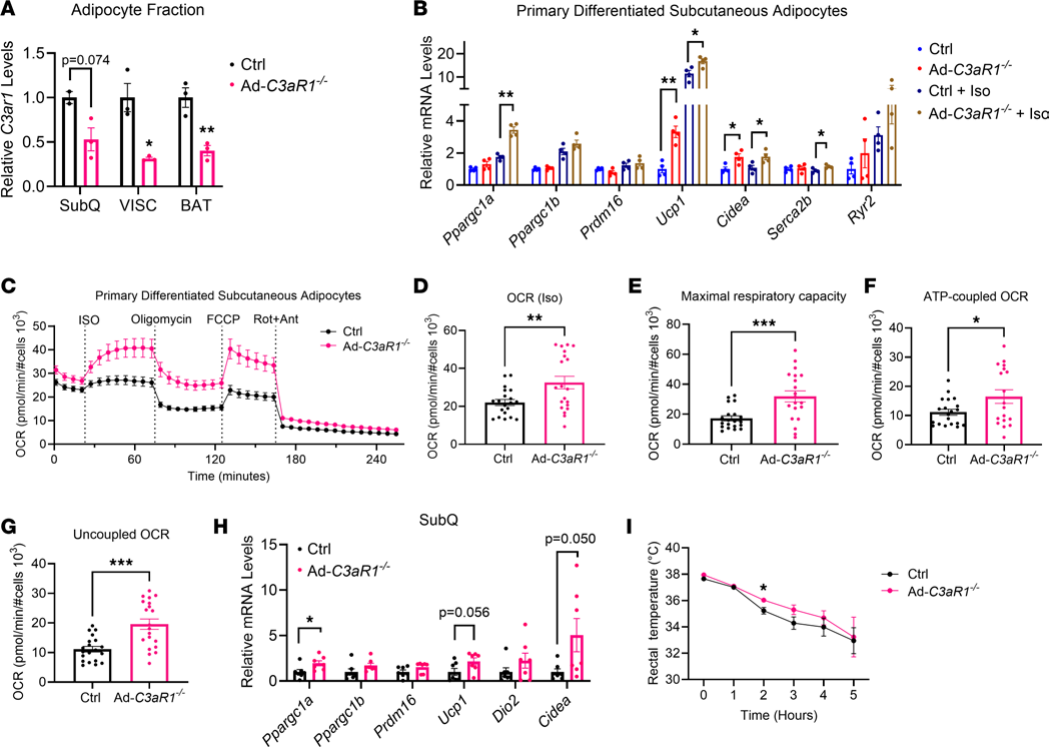
Adipocyte-specific *C3aR1*-KO male mice and adipose thermogenic capacity in vitro and in vivo. (**A**) *C3aR1* gene expression in the adipocyte fraction of 7-week-old control and Ad-*C3aR1^–/–^* male mice fed a regular diet at ambient temperature. *n* = 2–3/group. (**B**) Thermogenic gene expression in primary SubQ adipocytes treated with Iso from control and Ad-*C3aR1^–/–^* male mice. *n* = 4/group. (**C**) Oxygen consumption rate (OCR) of primary control and Ad-*C3aR1^–/–^* SubQ adipocytes. *n* = 18–20/group. FCCP, carbonyl cyanide 4-(trifluoromethoxy) phenylhydrazone; Rot, rotenone; Ant, antimycin. (**D**–**G**) Quantification of Iso-stimulated OCR (**D**), maximal respiratory capacity (**E**), ATP-coupled OCR (**F**), and uncoupled OCR (**G**) from primary control and Ad-*C3aR1^–/–^* SubQ adipocytes. (**H**) Thermogenic gene expression in SubQ fat of 10- to 12-week-old control and Ad-*C3aR1^–/–^* male mice fed a regular diet at ambient temperature. *n* = 7/group. (**I**) Body temperature of 10- to 12-week-old control and Ad-*C3aR1^–/–^* male mice during acute cold exposure. *n* = 7–10/group. Data are presented as mean ± SEM. Unpaired 2-tailed *t* test is used for comparison. *P < 0.05, **P < 0.01, ***P < 0.001.

To directly interrogate the role of C3aR1 on adipocytes in adaptive thermogenesis, we generated adipocyte-specific *C3aR1*-KO mice by crossing *C3ar1*-floxed mice with adiponectin-Cre BAC transgenic mice (Ad-*C3aR1*^–/–^). Because of the cellular heterogeneity in adipose tissues with macrophages and immune cells expressing high levels of C3aR1, we analyzed *C3ar1* gene expression in the adipocyte fraction to confirm the adipocyte-specific KO in this system. Our results showed 50%–65% knockdown of *C3ar1* in adipocytes in Ad-*C3aR1*^–/–^ compared with controls (Figure 3A). SubQ adipocytes from male Ad-*C3aR1*^–/–^ mice showed a robust 4-fold increase in *Ucp1* and 2-fold increase in *Cidea* compared with controls (Figure 3B). Stimulation of these cells with isoproterenol further augmented thermogenic gene expression, especially in adipocytes lacking C3aR1 (Figure 3B). The increased expression of thermogenic genes in *C3aR1*-KO SubQ adipocytes prompted us to investigate the respiratory activity of isolated adipocytes from control and adipocyte-specific *C3aR1*-KO mice. SubQ adipocytes from Ad-*C3aR1*^–/–^ mice displayed double the maximal respiratory capacity and 50% more isoproterenol-stimulated respiration that was mostly due to uncoupled respiration but also with elevated ATP-coupled respiration (sensitive to oligomycin) compared with controls (Figure 3, C–G). In the SubQ adipose tissue, *Ppargc1a* was increased with similar trends in *Ucp1* and *Cidea* in male Ad-*C3aR1*^–/–^ mice (Figure 3H). In contrast, brown adipocytes from control and Ad-*C3aR1*^–/–^ mice did not show any significant differences in thermogenic gene expression or mitochondrial respiration in response to isoproterenol (Supplemental Figure 3, A–F). There were also no differences in thermogenic gene expression between control and Ad-*C3aR1*^–/–^ brown and VISC adipose tissues in male mice (Supplemental Figure 3, G and H). UCP1 protein levels were also not affected in male Ad-*C3aR1*^–/–^ brown and SubQ fat compared with controls (Supplemental Figure 3, I and J). These results suggest that C3aR1 on adipocytes regulates thermogenic gene expression and mitochondrial respiration mostly in SubQ but not brown adipocytes.

Given our findings of enhanced thermogenesis in SubQ but not brown adipocytes in the Ad-*C3aR1*^–/–^ mice, we challenged male mice to cold and observed a modest improvement in cold tolerance in the KO mice (Figure 3I). There was no difference in body weight between the 2 groups before and after the cold challenge (Supplemental Figure 3K). We further measured heat production directly from the SubQ and brown fat ex vivo using calScreener. We found a mild trend toward increased heat production in the brown but not SubQ fat of male Ad-*C3aR1*^–/–^ mice compared with controls (Supplemental Figure 3, L and M). Together, these results suggest a mild effect of improved adipose thermogenesis and cold tolerance with *C3ar1* ablation in adipocytes.

### Sex-associated differences in the alternative complement pathway.

**Figure 4:**
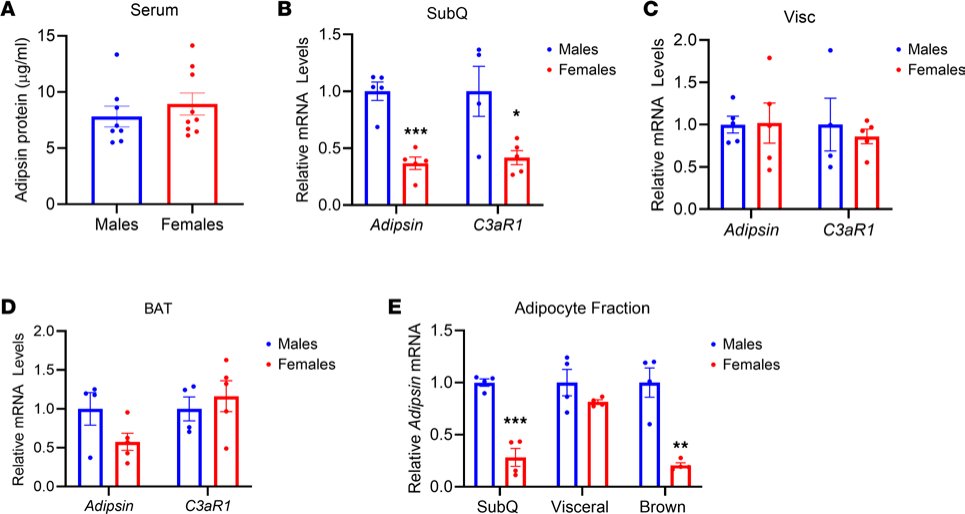
Sex-dependent differences in alternative complement pathway components. (**A**) ELISA for adipsin from serum of male and female WT mice at 10–11 weeks old on regular diet. *n* = 8/group. (**B**–**D**) Relative *Adipsin* and *C3aR1* gene expression in SubQ (**B**), VISC (**C**), and brown (**D**) adipose tissue of 10- to 11-week-old male and female WT mice on regular diet. *n* = 4–5/group. (**E**) Relative *Adipsin* gene expression in the adipocyte fraction of 10- to 11-week-old male and female WT mice on regular diet. *n* = 4/group. Data are presented as mean ± SEM. Unpaired 2-tailed *t* test is used for comparison. *P < 0.05, **P < 0.01, ***P < 0.001.

Clinical studies have illuminated sex differences in alternative complement pathway activity but not in the classical and lectin pathways (30). The circulating levels of 2 important alternative complement components, C3 and adipsin, show sex-dependent differences (30, 31). These sex differences in humans prompted us to investigate if there is a similar sexual dimorphism in mice. We found no difference in circulating adipsin levels between male and female mice (Figure 4A). Since adipsin is predominantly secreted by adipose tissues, we assayed *Adipsin* and *C3aR1* levels in adipose tissues of male and female mice. We found that *Adipsin* mRNA level was lower in the thermogenic adipose tissue of female mice, especially in SubQ adipose tissue (Figure 4, B–D). However, *C3aR1* was only decreased in the SubQ adipose tissue of female mice compared with males (Figure 4B). *C3aR1* gene expression was not different between female and male mice in the VISC adipose tissue and BAT (Figure 4, C and D). Next, we assessed *Adipsin* mRNA levels in the adipocyte fractions from the different major fat depots. Our results showed that *Adipsin* was significantly lower in SubQ and brown adipocytes of female mice compared with males (Figure 4E). These results reveal that there is sexual dimorphism in *Adipsin* expression in SubQ and brown but not VISC adipocytes in mice.

### Ad-C3aR1^–/–^ female mice display decreased thermogenic gene expression and cold intolerance.

**Figure 5:**
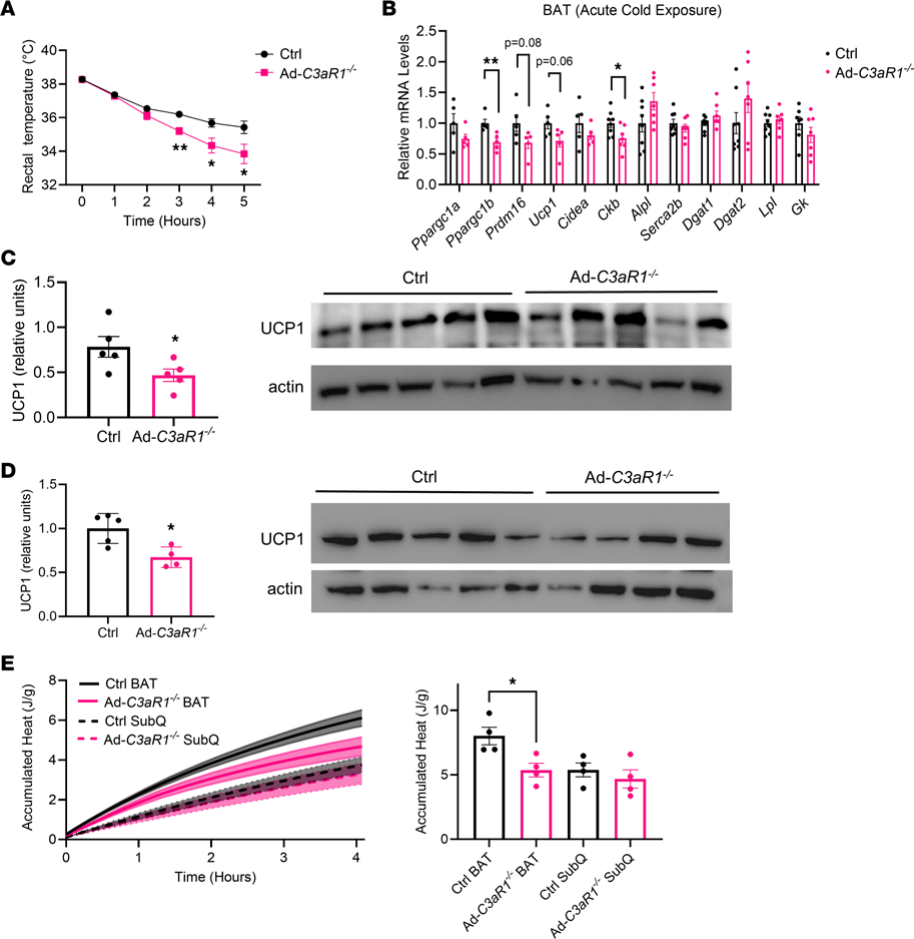
Ad-*C3aR1^–/–^* female mice have impaired thermogenic capacity and are cold intolerant. (**A**) Body temperature of control and Ad-*C3aR1^–/–^* female mice during acute cold exposure. *n* = 5/group. (**B**) Thermogenic gene expression in BAT of 10- to 12-week-old control and Ad-*C3aR1^–/–^* female mice following an acute (6 hours) cold exposure. *n* = 5–8/group. (**C**) Brown fat UCP1 protein levels normalized to actin by Western blot in control and Ad-*C3aR1^–/–^* female mice at ambient temperature. *n* = 5/group. (**D**) SubQ fat UCP1 protein levels normalized to actin by Western blot in control and Ad-*C3aR1^–/–^* female mice at ambient temperature. *n* = 4–5/group. (**E**) Accumulated heat in joules recorded from wells in duplicates containing adipose tissue from control and Ad-*C3aR1^–/–^* female mice fed a regular diet at ambient temperature. *n* = 4/group. Data are presented as mean ± SEM. Unpaired 2-tailed *t* test is used for comparison. *P < 0.05, **P < 0.01.

Because we observed differences in expression of alternative pathway components between male and female mice, we tested if adipocyte C3aR1 expression may have different effects in female mice. In stark contrast with male mice, Ad-*C3aR1*^–/–^ female mice exhibited substantial impairments in defending their core body temperature during acute cold stress compared with controls, without any changes in body weights (Figure 5A and Supplemental Figure 4A). We found that *Ppargc1b* and creatine kinase B (*Ckb*) were mildly decreased, with similar trends for *Prdm16* and *Ucp1*, in the BAT of Ad-*C3aR1^–/–^* female mice compared with the control group during acute cold stress (Figure 5B). At room temperature, there was a similar trend of lower *Ucp1* in the BAT of Ad-*C3aR1^–/–^* female mice compared with controls (Supplemental Figure 4B). Thermogenic gene expression was not lower, with *Serca2b* and *Ryr2* being elevated in the SubQ fat of Ad-*C3aR1^–/–^* compared with controls during acute cold exposure (Supplemental Figure 4C). Brown and SubQ fat UCP1 protein was also approximately 30% lower in Ad-*C3aR1^–/–^* female KO mice compared with controls (Figure 5, C and D). Importantly, we observed 30% less heat production in the BAT without changes in SubQ fat of Ad-*C3aR1*^–/–^ female mice compared with controls (Figure 5E and Supplemental Figure 4D). These results suggest that impaired BAT thermogenesis in Ad-*C3aR1*^–/–^ female mice substantially compromised their endothermic response to acute cold exposure. We did not detect any gross differences in brown adipocyte morphology and lipid stores in the BAT between the 2 groups (Supplemental Figure 4E). Consistent with the changes in thermogenic gene expression, UCP1 staining was also fainter in the BAT of female Ad-*C3aR1*^–/–^ mice compared with controls (Supplemental Figure 4F). Differentiated SubQ adipocytes from female Ad-*C3aR1*^–/–^ mice did not show the same gene expression phenotype (Supplemental Figure 4G) in vitro as male Ad-*C3aR1*^–/–^ adipocytes (Figure 3B), suggesting cell-intrinsic differences between sexes. In vitro–differentiated brown adipocytes from female Ad-*C3aR1*^–/–^ mice did not exhibit the enhanced thermogenic gene expression seen in vivo with the BAT in female Ad-*C3aR1*^–/–^ mice, suggesting critical in vivo inputs (Supplemental Figure 4H). Collectively, these results indicate that adipocyte C3aR1 in female mice is critical for adaptive thermogenesis in the classical brown fat and defense against cold.

## Discussion

Adipose tissues can synthesize the major components of the alternative complement pathway and are one of the targets of complement activation in an autocrine or paracrine fashion (23). Although alternative complement activation is thought to promote the pathological progression of obesity-related metabolic diseases, the precise physiological mechanisms linking alternative complement pathways to metabolic dysfunction are not well defined. Here, we provide an essential role of the alternative complement adipsin/C3a/C3aR1 signaling axis in the cold-induced browning of white adipocytes. Deletion of *Adipsin* or *C3ar1* increases SubQ adipocyte thermogenic gene expression, resulting in adipocyte browning in cold-exposed male mice. SubQ adipocytes lacking C3aR1 also demonstrate higher respiration rates, though it remains to be determined which thermogenic pathway or combination thereof is responsible for the phenotype, including Serca2b/Ryr2 and glycolytic pathways that were mildly changed in some of our studies (32, 33). With UCP1 deficiency, UCP1-independent pathways can compensate (34, 35). It is also important to note that our studies tested C3aR1 KO but not in combination with UCP1, CKB, or tissue nonspecific alkaline phosphatase KO (36, 37).

We demonstrate sexual dimorphism in the expression of *Adipsin* and *C3ar1*, key alternative complement components in adipose tissues. When and how the sex differences in gene expression manifest are important questions worthy of future research. It is unknown if the sex-biased gene expression arises early during development, is dependent on sex hormones, or may be cell type or organ specific. Critically, the effects of adipocyte-specific C3aR1 deficiency on adipocyte browning are diametrically opposite between male and female mice. Here, we provide a rare molecular example of a non–sex hormone that regulates thermogenic adipose function in a sex-dependent fashion. Although it is unclear how much sex differences have been ignored in the past, our study highlights the importance of assessing for potential sexual dimorphism in adipose biology. C3a/C3aR1 seems to inhibit browning in males while stimulating adipocyte thermogenesis in female mice. This could be due to different tonicity of C3aR1 signaling between sexes, other external inputs, and/or the intrinsic differences in the C3aR1 signaling pathway between male and female beige adipocytes. Notably, we observe differences between *Adipsin^–/–^* and Ad-*C3ar1^–/–^* female mice, with only the latter showing cold intolerance. A likely explanation for the discrepancy is that *Adipsin^–/–^* mice still express C3aR1, and there are other ligands for C3aR1 (e.g., TLQP-21) and other means of generating C3a, such as with C3 tickover. The functional contributions of each of the thermogenic pathways whether UCP1 dependent or UCP1 independent in beige or brown adipocytes are outstanding questions for the field. How the downstream C3aR1 signaling pathway in adipocytes may differ between male and female mice will be of great future interest. It is noteworthy that autoimmune complement diseases in humans also display sexual dimorphism (31).

External stimuli can be leveraged to offset metabolic diseases by activating thermogenesis in brown and beige adipose tissues (38). Adipsin is the rate-limiting enzyme in the alternative pathway and controls downstream products such as C3a, C3b, and C5a (39). Our data suggest that SubQ beige and brown adipocytes are a major target of adipsin/C3a through C3aR1. During complement activation, adipsin catalyzes the formation of the C3 convertase. C3 is highly abundant, but C3a is quickly converted by carboxypeptidase B or N into the inactive C3a-desArg45 that does not signal through C3aR1 (20, 40). Indeed, the data here suggest that *C3aR1* deficiency markedly upregulated thermogenesis and mitochondrial respiration in male SubQ adipocytes. On the other hand, C3a/C3aR1 signaling is also involved in macrophage-mediated inflammation (25). Alternatively activated macrophages and eosinophils have also been shown to enhance adipocyte browning and protect against diet-induced insulin resistance (41, 42). Therefore, it would be of future interest to determine whether C3a/C3aR1 signaling is involved in macrophage-related adipocyte browning in adipose tissues.

Similarly, a study utilizing whole-body C3aR1 and C5aR1 single- and double-KO mice reported increased adipose browning in male receptor-KO mice (43). The postulated mechanism involved regulatory T cells and browning via the inosine/A2aR pathway. However, the authors only utilized male mice, with general results of adipose browning consistent with this study. Clinical studies have demonstrated sex differences in alternative complement activity and complement component levels in healthy Caucasians (30). Alternative pathway activity is higher in males than in females (30). Circulating levels of C3 are higher in males, while adipsin levels are higher in females (30). Other clinical studies have also confirmed that C3 has higher expression in the cerebrospinal fluid and plasma of men than women (31, 44). Our results show for the first time to our knowledge that in C57BL/6J mice, circulating levels of adipsin do not differ by sex, but its gene expression is higher in WAT and thermogenic adipocytes in males than in females. These results suggest that biological sex should be taken into account in alternative complement–related pathology as well as in complement-targeted therapies. Both genetics (such as the X chromosome) and hormonal differences are known to explain the effects of sexual dimorphism on immunity. But what is causing the difference in the key complement components in the alternative pathway is still unclear. Several clues have suggested that sex may form a possible confounding factor in complement-mediated diseases. For example, in one age-related macular degeneration study, significant differences in alternative pathway component levels were found between the sexes (45). Therefore, further research is needed to elucidate the importance of sexual dimorphism on the complement system. In the future, it will be critical to demonstrate how sex hormones affect the complement system by administrating estrogen and testosterone to healthy and castrated mice of both sexes. Alternative complement levels and activity can also be studied in human study participants receiving hormone replacement therapy.

Although complement factors are best known for treating autoimmune diseases, their effects on adipose thermogenesis are new to the adipose metabolism field. Our analysis reveals that adipocyte-specific KO of *C3aR1* mice results in major sex differences in response to cold stimulation. In particular, female mice with *C3aR1* KO in adipocytes exhibit pronounced cold intolerance. We do not yet have a mechanistic understanding of the downstream signaling pathway of C3aR1 in regulating the thermogenic gene program in adipocytes and why the results are discrepant between males and females. This may include differences in mitochondrial mass or activity and/or sensitivity to acute and chronic thermogenic stimuli. To our knowledge, this is the first demonstration of sexual dimorphism for brown and beige adipocytes where a molecular receptor shows diametrically opposed functions between sexes. Previous studies have also found sexual dimorphism in the effects of adipose thermogenesis or thermogenesis-related markers (40, 46). Filatov et al. (46) found that the thermogenic protein UCP1 was induced by cold exposure at the mRNA and protein levels in male Pacap-null mice; however, UCP1 protein induction was attenuated after cold adaptation in female Pacap-null mice. The observed sex-specific differences in the effects of C3aR1 on fat thermogenesis may be related to estrogen. There is evidence for sexual dimorphism in regulation of BAT function and adipose tissue browning in rodent models and humans (47, 48). For example, Benz et al. demonstrated that estrogen receptor α signaling in adipocytes can directly activate lipolysis (49). In future studies, it will be of interest to determine if adipocyte C3aR1 is involved in estrogen receptor α signaling–related lipolysis in adipocytes. The mechanistic relationship between estrogen and complement factors remains to be understood with potential for crosstalk between the pathways. Our study highlights that the physiological response to cold stress is regulated differently between the sexes and stresses the importance of analyzing both sexes when assessing mechanisms of energy homeostasis and adaptive thermogenesis in fat.

## Methods

### Sex as a biological variable.

Our study examined male and female animals, and sex-dimorphic effects are reported.

### Mice.

WT and *Adipsin^–/–^* mice were backcrossed to C57BL/6J background as described previously (21). Whole-body C3aR1-KO mice were purchased from The Jackson Laboratory (strain 005712). *C3ar1*-floxed mice are on the C57BL/6J background (gift from Peter S. Heeger, Cedars-Sinai, Los Angeles, California, USA) as described (50). Adiponectin-Cre BAC transgenic mice were purchased from The Jackson Laboratory (strain 028020). *C3ar1*-floxed homozygous mice were used in the experiments as controls from the same backcross generation. All mice were maintained in plastic cages under a 12-hour light/12-hour dark cycle at constant temperature (22°C) with free access to water and food. For the diet-induced obesity model, mice were fed a 60% HFD (D12492i, Research Diets) for 16 weeks.

### Cold-induced thermogenesis.

All the experimental mice were placed in a cold chamber (4°C) for 6 hours (acute cold exposure) or 1 week (chronic cold exposure) with free access to water and food. During acute cold exposure, rectal body temperature was measured using a BAT-12 Microprobe Thermometer (Physitemp).

### Indirect calorimetry.

Metabolic rate was measured by indirect calorimetry in a metabolic cage, a component of the Comprehensive Lab Animal Monitoring System (Columbus Instruments). Mice were housed individually and maintained at 22°C under a 12-hour light/12-hour dark cycle. Food and water were available ad libitum.

### Histological analysis.

The adipose tissue was immediately perfused with PBS and fixed with 10% neutral-buffered formalin (VWR). Adipose tissues were then transferred to 70% ethanol. Paraffin-embedding, sectioning, and H&E staining were done by the Memorial Sloan Kettering Cancer Center Zuckerman Research Center Laboratory of Comparative Pathology core facility. For UCP1 immunohistochemistry, slides were dewaxed in xylene; hydrated in 100%, 95%, 80%, and 70% ethanol; and rinsed in water, and antigen retrieval was performed in 10 mM sodium citrate buffer (pH = 6.0) by boiling sections. Quenching of endogenous peroxidases was performed using 3% H_2_O_2_ solution (VWR). Slides were incubated with rabbit polyclonal UCP1 antibody (Abcam, ab10983) overnight at 4°C. Slides were washed in PBS with Tween 20 followed by incubation with HRP-conjugated secondary antibody (goat anti-rabbit, VWR/Jackson ImmunoResearch, 111-035-144). Then avidin–biotin peroxidase complex (VECTASTAIN ABC kit, PK-7200, Vector Laboratories) was added for 30 minutes. Slides were developed using 3,3′-diaminobenzidine or 3-amino-9-ethyl carbazole (MilliporeSigma) and counterstained with hematoxylin.

### In vitro differentiation of primary adipocytes.

For primary adipocytes, stromal vascular fraction from inguinal or VISC adipose from 6- to 7-week- old mice was prepared and differentiated for 6–8 days. Primary white adipocytes were cultured in DMEM/F12K media (Gibco, Thermo Fisher Scientific) with 10% fetal bovine serum (FBS) at 37°C, 5% CO_2_, until confluent. White adipocytes were differentiated via a 48-hour treatment with 0.5 mM 3-isobutyl-1-methylxanthine (IBMX), 1 mM dexamethasone, 850 nM insulin, and 1 mM rosiglitazone, followed by 48 hours with 850 nM insulin and 1 mM rosiglitazone, then a further 48 hours with 850 nM insulin. Primary brown adipocytes were cultured in DMEM/F12K with 10% FBS at 37°C, 5% CO_2_, until confluent. Brown adipocytes were differentiated via a 48-hour treatment with 0.5 mM IBMX, 5 μM dexamethasone, 1 nM T3, 2 μM tamoxifen, 400 nM insulin, and 1 μM rosiglitazone, followed by 48 hours with 400 nM insulin and 1 μM rosiglitazone, and a further 48 hours with 400 nM insulin. The fully differentiated adipocytes were treated with isoproterenol (10 or 100 nM) for 6 hours.

### Seahorse assay.

Primary SubQ adipocytes and brown adipocytes were plated into 96-well cell culture microplates (Agilent), and cells were plated in Seahorse XF base medium (Agilent) containing 2 mM l-glutamine and 5 mM glucose adjusted to pH 7.4. The Seahorse microplate was incubated without supplemental CO_2_ at 37°C for 1 hour before assay. Oxygen consumption rates and extracellular acidification rates were measured using an XFe96 Seahorse (Agilent). During the assay 0.5 μM rotenone and antimycin, 1 μM isoproterenol, 3 μM oligomycin, and 2 μM carbonyl cyanide 4-(trifluoromethoxy)phenylhydrazone (FCCP) were injected.

### RNA extraction and real-time quantitative PCR.

Total RNA from adipose and adipocytes was isolated using the RNeasy Mini Kit (QIAGEN) per manufacturer’s protocol. A total of 1 μg RNA was reverse-transcribed using high-capacity cDNA RT kit (Thermo Fisher Scientific). Quantitative PCR was performed using the SYBR Green Master Mix (Quanta) and specific gene primers on the QuantStudio 6 Flex system (Thermo Fisher Scientific). Relative mRNA levels were determined by normalizing to ribosomal protein S18 (Rps18) levels using the ΔΔC_T_ method. Primer sequences are listed in Supplemental Table 1. Approximate C_T_ values for certain genes are listed in Supplemental Table 2.

### Western blot.

Adipose tissues were homogenized in RIPA buffer with protease and phosphatase inhibitor cocktail. Clarified protein extracts were obtained by multiple centrifugation steps for 10 minutes at 13,400*g* at 4°C. Protein concentration was measured using bicinchoninic acid assay (Pierce, Thermo Fisher Scientific). Protein extracts were resolved on a NuPAGE Bis-Tris gel (Thermo Fisher Scientific) and transferred to a PVDF membrane. Membranes were incubated overnight at 4°C with appropriate primary antibodies. Anti-UCP1 was from Abcam (ab10983), and anti–β-actin was from Thermo Fisher Scientific (MA515739HRP). Detection of proteins was carried out by incubations with HRP-conjugated secondary antibodies described above followed by enhanced chemiluminescence detection reagents. Band density was quantified using Fiji/ImageJ (NIH).

### Serum adipsin measurements.

For measuring mouse serum adipsin levels, blood was collected from the tail vein, the serum was separated by centrifugation for 10 minutes at 3,000*g* at 4°C, and then adipsin levels were measured by ELISA (R&D Systems, Bio-Techne) following the manufacturer’s protocol.

### Isothermal microcalorimeter assay.

For heat detection experiments, we used the calScreener, a 48-channel isothermal microcalorimeter (Symcel), with its corresponding 48-well plate (calPlate). Each well consists of a screw-capped titanium vial. Data were continuously collected with the corresponding calView software (Version 1.0.33.0, 2016, Symcel). For the assays, the machine was set and calibrated at 37°C. General handling and device manipulation were done according to the manufacturer’s recommendations. We added 400 μL DMEM/F12K media with 10% FBS and around 30–40 mg adipose tissue in each vial. The calView software was used for the calScreener isothermal microcalorimetry (IMC) data collection and analysis. The direct measurement in IMC is heat flow (power in J/s) as a function of time. The heat flow gives the kinetic behavior and response of the sample over time. Data can also be expressed as the total accumulated heat (energy expressed in joules) over time as an alternative data presentation of the cellular response to treatment.

### Statistics.

All statistical analyses were performed using GraphPad Prism 9. Unpaired 2-tailed *t* tests, unpaired Welch’s *t* test, and 2-way ANOVA were used. *P* < 0.05 was considered statistically significant.

### Study approval.

All animal studies were approved by the Institutional Animal Care and Use Committee and Research Animal Resource Center at Weill Cornell Medical College.

### Data availability.

The main data supporting the findings of this study are available within the article and its supplemental files, including the Supporting Data Values.

## Author contributions

LM and JCL designed the study and wrote the manuscript with input from all authors. LM, AG, ARN, EC, SMR, and AL performed and analyzed the animal experiments. LM, AG, and ARN developed and analyzed the in vitro experiments. SMR and LT provided scientific input. JCL conceived and supervised the study.

## Supplementary Material

Supplemental data

Unedited blot and gel images

Supplemental tables 1-2

Supporting data values

## Version 1 05/07/2024

In-Press Preview

## Version 2 06/10/2024

Electronic publication

## References

[1] Powell-Wiley TM (2021). Obesity and cardiovascular disease: a scientific statement from the American Heart Association. Circulation.

[2] Gonzalez-Muniesa P (2017). Obesity. Nat Rev Dis Primers.

[3] Yanovski SZ, Yanovski JA (2014). Long-term drug treatment for obesity: a systematic and clinical review. JAMA.

[4] Rosenstock J (2021). Efficacy and safety of a novel dual GIP and GLP-1 receptor agonist tirzepatide in patients with type 2 diabetes (SURPASS-1): a double-blind, randomised, phase 3 trial. Lancet.

[5] Rizvi AA, Rizzo M (2022). The emerging role of dual GLP-1 and GIP receptor agonists in glycemic management and cardiovascular risk reduction. Diabetes Metab Syndr Obes.

[6] Chouchani ET (2019). New advances in adaptive thermogenesis: UCP1 and beyond. Cell Metab.

[7] Cannon B, Nedergaard J (2004). Brown adipose tissue: function and physiological significance. Physiol Rev.

[8] Cypess AM (2013). Anatomical localization, gene expression profiling and functional characterization of adult human neck brown fat. Nat Med.

[9] Cohen P, Kajimura S (2021). The cellular and functional complexity of thermogenic fat. Nat Rev Mol Cell Biol.

[10] Wu J (2012). Beige adipocytes are a distinct type of thermogenic fat cell in mouse and human. Cell.

[11] Lee YH (2015). Cellular origins of cold-induced brown adipocytes in adult mice. FASEB J.

[12] Long JZ (2014). A smooth muscle-like origin for beige adipocytes. Cell Metab.

[13] Pollard AE, Carling D (2020). Thermogenic adipocytes: lineage, function and therapeutic potential. Biochem J.

[14] Shao M (2019). Cellular origins of beige fat cells revisited. Diabetes.

[15] Sakers A (2022). Adipose-tissue plasticity in health and disease. Cell.

[16] Cypess AM (2009). Identification and importance of brown adipose tissue in adult humans. N Engl J Med.

[17] Van Marken Lichtenbelt WD (2009). Cold-activated brown adipose tissue in healthy men. N Engl J Med.

[18] Virtanen KA (2009). Functional brown adipose tissue in healthy adults. N Engl J Med.

[19] Dunkelberger JR, Song WC (2010). Complement and its role in innate and adaptive immune responses. Cell Res.

[20] Mamane Y (2009). The C3a anaphylatoxin receptor is a key mediator of insulin resistance and functions by modulating adipose tissue macrophage infiltration and activation. Diabetes.

[21] Lo JC (2014). Adipsin is an adipokine that improves β cell function in diabetes. Cell.

[22] Shim K (2020). Complement activation in obesity, insulin resistance, and type 2 diabetes mellitus. World J Diabetes.

[23] Choy LN (1992). Adipsin and an endogenous pathway of complement from adipose cells. J Biol Chem.

[24] Gomez-Banoy N (2019). Adipsin preserves beta cells in diabetic mice and associates with protection from type 2 diabetes in humans. Nat Med.

[25] Lim J (2013). C5aR and C3aR antagonists each inhibit diet-induced obesity, metabolic dysfunction, and adipocyte and macrophage signaling. FASEB J.

[26] Wu J (2013). Adaptive thermogenesis in adipocytes: is beige the new brown?. Genes Dev.

[27] Trouw LA (2017). The complement system as a potential therapeutic target in rheumatic disease. Nat Rev Rheumatol.

[28] Zwarthoff SA (2018). Functional characterization of alternative and classical pathway C3/C5 convertase activity and inhibition using purified models. Front Immunol.

[29] Mayilyan KR (2012). Complement genetics, deficiencies, and disease associations. Protein Cell.

[30] Gaya da Costa M (2018). Age and sex-associated changes of complement activity and complement levels in a healthy caucasian population. Front Immunol.

[31] Kamitaki N (2020). Complement genes contribute sex-biased vulnerability in diverse disorders. Nature.

[32] Chen Y (2019). Thermal stress induces glycolytic beige fat formation via a myogenic state. Nature.

[33] Ikeda K (2017). UCP1-independent signaling involving SERCA2b-mediated calcium cycling regulates beige fat thermogenesis and systemic glucose homeostasis. Nat Med.

[34] Oeckl J (2022). Loss of UCP1 function augments recruitment of futile lipid cycling for thermogenesis in murine brown fat. Mol Metab.

[35] Rahbani JF (2024). Parallel control of cold-triggered adipocyte thermogenesis by UCP1 and CKB. Cell Metab.

[36] Rahbani JF (2021). Creatine kinase B controls futile creatine cycling in thermogenic fat. Nature.

[37] Sun Y (2021). Mitochondrial TNAP controls thermogenesis by hydrolysis of phosphocreatine. Nature.

[38] Pfeifer A, Hoffmann LS (2015). Brown, beige, and white: the new color code of fat and its pharmacological implications. Annu Rev Pharmacol Toxicol.

[39] Forneris F (2010). Structures of C3b in complex with factors B and D give insight into complement convertase formation. Science.

[40] Campbell WD (2002). Inactivation of C3a and C5a octapeptides by carboxypeptidase R and carboxypeptidase N. Microbiol Immunol.

[41] Molofsky AB (2013). Innate lymphoid type 2 cells sustain visceral adipose tissue eosinophils and alternatively activated macrophages. J Exp Med.

[42] Wu D (2011). Eosinophils sustain adipose alternatively activated macrophages associated with glucose homeostasis. Science.

[43] Kong LR (2023). Loss of C3a and C5a receptors promotes adipocyte browning and attenuates diet-induced obesity via activating inosine/A2aR pathway. Cell Rep.

[44] Ritchie RF (2004). Reference distributions for complement proteins C3 and C4: a practical, simple and clinically relevant approach in a large cohort. J Clin Lab Anal.

[45] Silva AS (2012). Plasma levels of complement proteins from the alternative pathway in patients with age-related macular degeneration are independent of Complement factor H Tyr^402^His polymorphism. Mol Vis.

[46] Filatov E (2021). Contribution of thermogenic mechanisms by male and female mice lacking pituitary adenylate cyclase-activating polypeptide in response to cold acclimation. Am J Physiol Endocrinol Metab.

[47] Frank AP (2018). Do estrogens enhance activation of brown and beiging of adipose tissues?. Physiol Behav.

[48] Kaikaew K (2021). Sex differences in brown adipose tissue function: sex hormones, glucocorticoids, and their crosstalk. Front Endocrinol (Lausanne).

[49] Benz V (2012). Sexual dimorphic regulation of body weight dynamics and adipose tissue lipolysis. PLoS One.

[50] Cumpelik A (2021). Dynamic regulation of B cell complement signaling is integral to germinal center responses. Nat Immunol.

