# Supplementary material for: Adipsin and adipocyte-derived C3aR1 regulate thermogenic fat in a sex-dependent fashion
Source: JCI Insight. 2024 May 7;9(11):e178925. doi: 10.1172/jci.insight.178925 (PMC11382875; doi:10.1172/jci.insight.178925)
Supplement: Supplemental data [file jciinsight-9-178925-s006.pdf]

SUPPLEMENTAL FIGURES

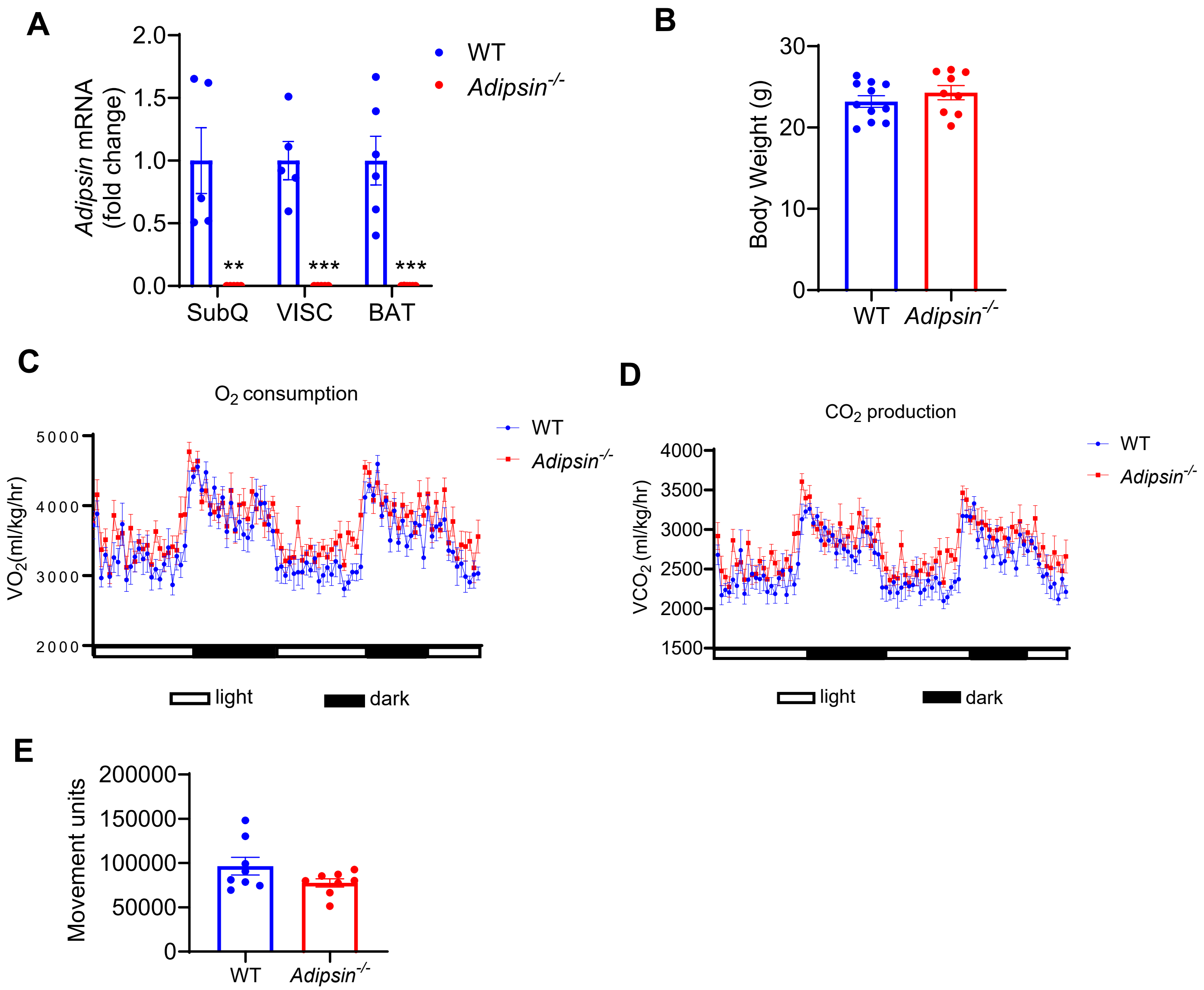

Supplemental Figure 1.

A) *Adipsin* mRNA expression in adipose tissues of 10-12 week old wild type (WT) and *Adipsin* knockout male mice fed a regular diet at ambient temperature. N=5-6/group.

B) Body weights of WT and *Adipsin* knockout male mice on 12 weeks of regular diet. N=9-11/group.

C and **D)** O_2 consumption (**C**) and CO_2 production (**D**) rates of WT and *Adipsin* knockout male mice were measured by indirect calorimetry after 4 weeks on high fat diet (HFD). N=8/group.

E) Locomotor activity of WT and *Adipsin* knockout male mice after 4 weeks of HFD measured in metabolic chambers. N=8/group.

Data are presented as mean± S.E.M. Unpaired t-test is used for comparison between groups in **A** and **B**. *p < 0.05, **p < 0.01, ***p < 0.001.

Supplemental Figure 2

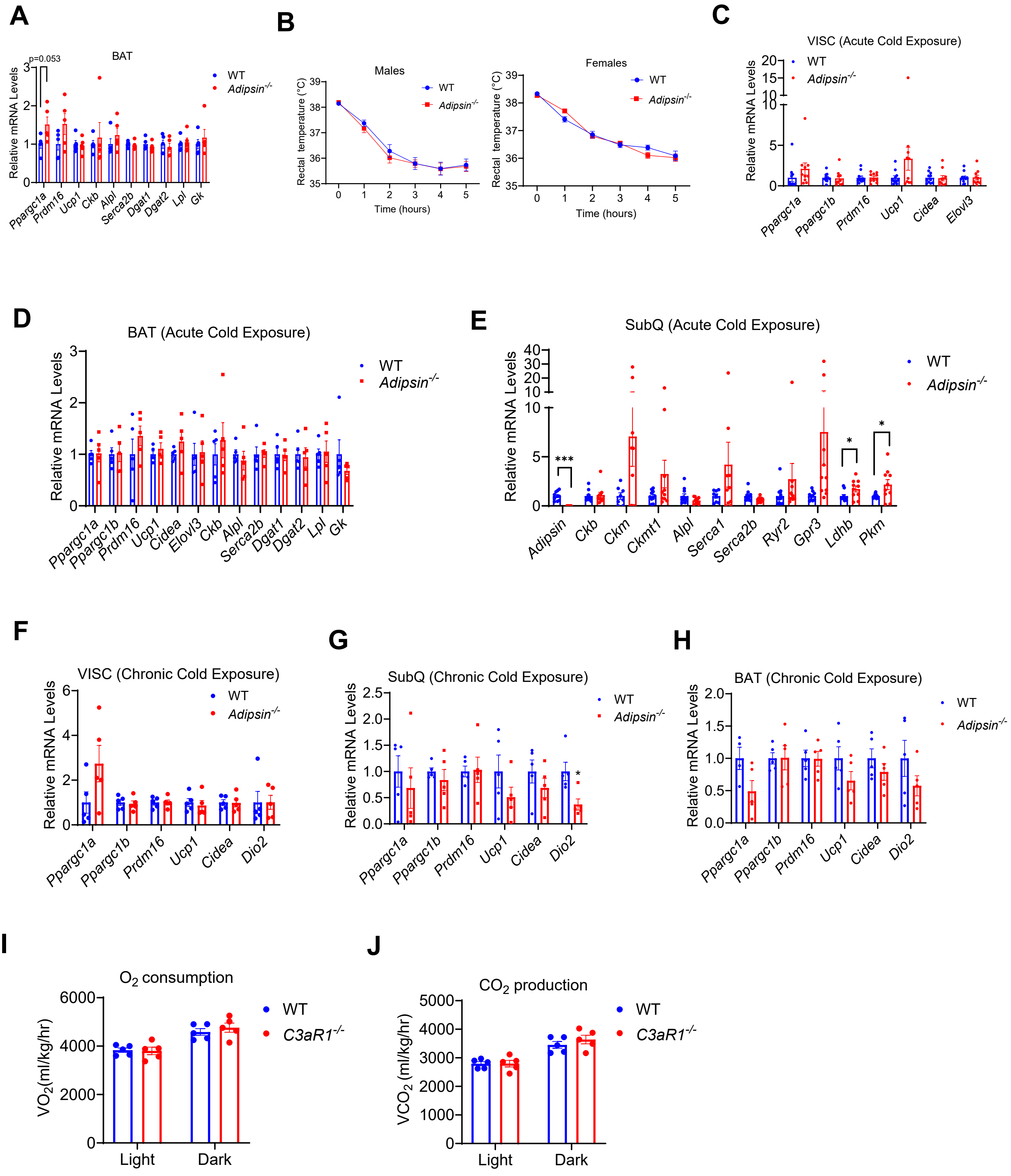

Supplemental Figure 2.

A) Thermogenic gene expression in brown adipose tissue (BAT) of 10-12 weeks old wild type (WT) and *Adipsin* knockout male mice fed a regular diet at ambient temperature. N=5/group.

B) Body temperature of 10-12 week old control and Ad-*C3aR1*$^{-/-}$ male and female mice during acute cold exposure. n=10-11/group for males and n=15-18/group for females.

C) Thermogenic gene expression in visceral (VISC) fat of 10-12 weeks old WT and *Adipsin* knockout male mice following an acute (6 hour) cold exposure. n=10-11/group.

D) Thermogenic gene expression in BAT of 10-12 week old WT and *Adipsin* knockout male mice following an acute (6 hour) cold exposure. n=5/group.

E) UCP1-independent thermogenic gene expression in subcutaneous (SubQ) fat of 10-12 week old WT and *Adipsin* knockout male mice following an acute (6 hour) cold exposure. n=8-11/group.

F-H) Thermogenic gene expression in VISC (**F**), SubQ (**G**) and brown (**H**) fat of 10-12 week old WT and *Adipsin* knockout male mice following a chronic (1 week) cold exposure. n=4-5/group.

I-J) O_2 consumption (**I**) and CO_2 production (**J**) rates of WT and *C3ar1* knockout mice were measured by indirect calorimetry after 4 weeks on high fat diet (HFD). n=5/group.

Data are presented as mean± S.E.M. Unpaired two-tailed t test is used for comparison. n.s., not significant. *p < 0.05, **p < 0.01, ***p < 0.001.

Supplemental Figure 3

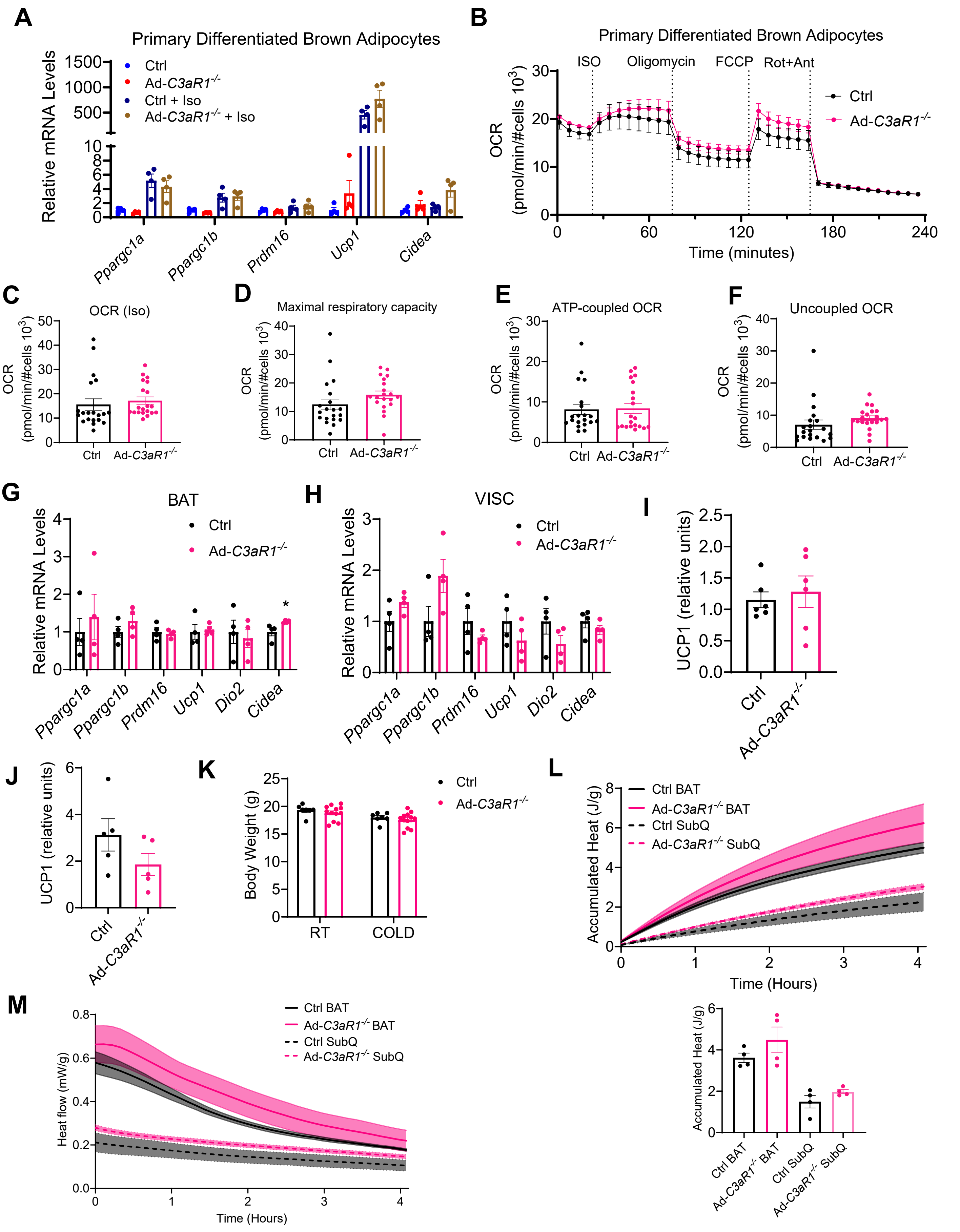

Supplemental Figure 3.

A) Thermogenic gene expression in primary brown adipocytes treated with isoproterenol from control and Ad-*C3aR1*$^{-/-}$ male mice. n=4/group.

B) OCR of primary control and Ad-*C3aR1* knockout brown adipocytes. n=20/group.

C-F) Quantification of isoproterenol-stimulated OCR (**C**), maximal respiratory capacity (**D**), Adenosine triphosphate (ATP)-coupled OCR (**E**), and uncoupled OCR (**F**) from primary control and Ad-*C3aR1* knockout brown adipocytes. n=20/group.

G and **H)** Thermogenic gene expression in brown (**G**) and visceral (VISC) (**H**) fat of 10-12 week old control and Ad-*C3aR1* knockout male mice fed a regular diet at ambient temperature. n=4/group.

I) Brown fat UCP1 protein levels normalized to actin by Western blot in control and Ad-*C3aR1*$^{-/-}$ male mice at ambient temperature**.** n=6/group.

J) Subcutaneous fat UCP1 protein levels normalized to actin by Western blot in control and Ad-*C3aR1*$^{-/-}$ male mice at ambient temperature**.** n=5/group.

K) Body weights of control and Ad-*C3aR1* knockout male mice at room temperature and 6 hours post cold exposure. n=7-12/group.

L) Accumulated heat in J recorded from wells in duplicates containing adipose tissue from control and Ad-*C3aR1*$^{-/-}$ male mice fed a regular diet at ambient temperature. n=4/group.

M) Heat flow in mW recorded from wells in duplicates containing adipose tissue from control and Ad-*C3aR1*$^{-/-}$ male mice fed a regular diet at ambient temperature. n=4/group.

Data are presented as mean$\pm$ S.E.M. Unpaired two-tailed t test is used for comparison.

Supplemental Figure 4

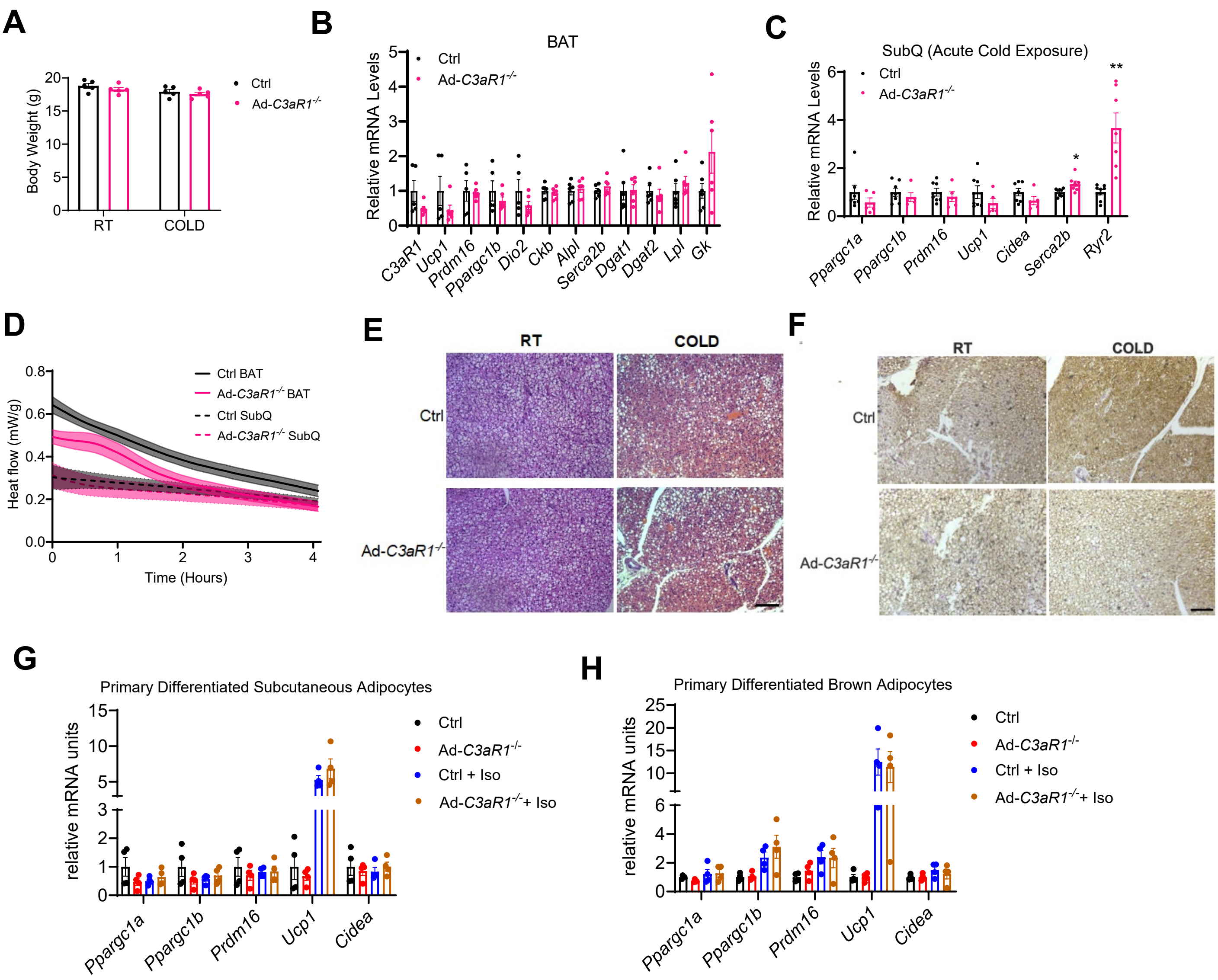

Supplemental Figure 4.

A) Body weight of control and Ad-*C3aR1* knockout female mice at room temperature and 6 hours post cold exposure. n=5/group.

B) Thermogenic gene expression in brown adipose tissue (BAT) of 10-12 week old control and Ad-*C3aR1*$^{-/-}$ female mice at room temperature. n=5-6/group.

C) Thermogenic gene expression of subcutaneous (SubQ) fat in 10-12 weeks WT and Ad-*C3aR1* knockout female mice following an acute (6 hour) cold exposure. n=5-8/group.

D) Heat flow in mW recorded from wells in duplicates containing adipose tissue from control and Ad-*C3aR1*$^{-/-}$ female mice fed a regular diet at ambient temperature. n=4/group.

E and **F)** Hematoxylin and eosin (**E**) and UCP1 (**F**) immunohistochemistry staining of brown adipose tissue (BAT) sections from 10-week-old control and Ad-*C3aR1* knockout female mice at room temperature (RT) and following acute cold exposure. Representative images are shown at 20x magnification. Scale bar, 200 μm.

G and **H)** Thermogenic gene expression in primary subcutaneous (**G**) and brown (**H**) adipocytes treated with isoproterenol (Iso) from control and Ad-*C3aR1*$^{-/-}$ female mice. n=4/group.

Data are presented as mean± S.E.M. Unpaired two-tailed t test is used for comparison. $*p < 0.05$, $**p < 0.01$.
