# Supplementary material for: Adipsin and adipocyte-derived C3aR1 regulate thermogenic fat in a sex-dependent fashion
Source: JCI Insight. 2024 May 7;9(11):e178925. doi: 10.1172/jci.insight.178925 (PMC11382875; doi:10.1172/jci.insight.178925)

Full unedited Blot: Female BAT for Figure 5C

IB: Ucp1

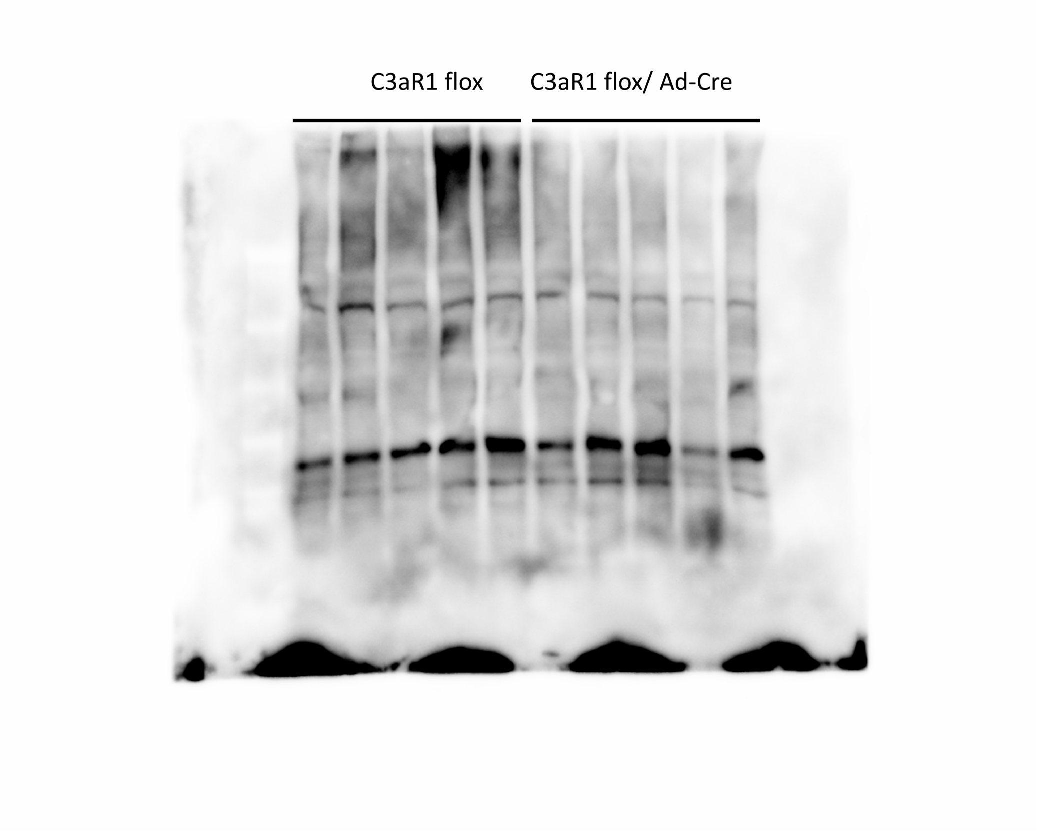

IB: Beta-actin

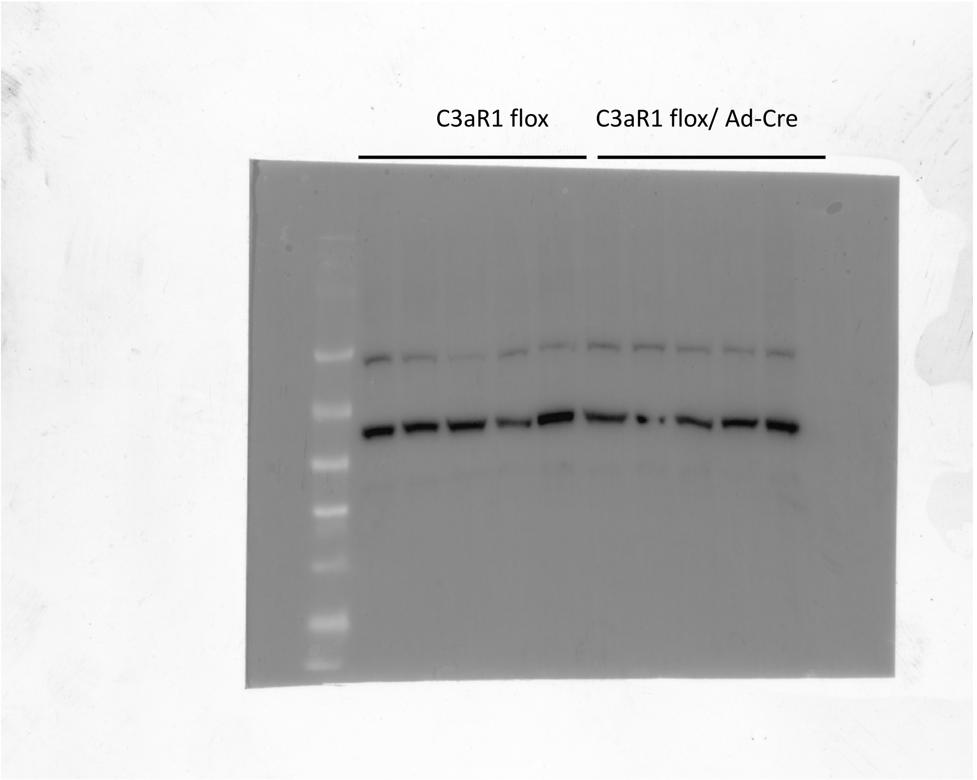

Full unedited Blot: Female SAT for Figure 5D

IB: Ucp1

IB: Beta-actin

C3aR1 flox C3aR1 flox/ Ad-Cre

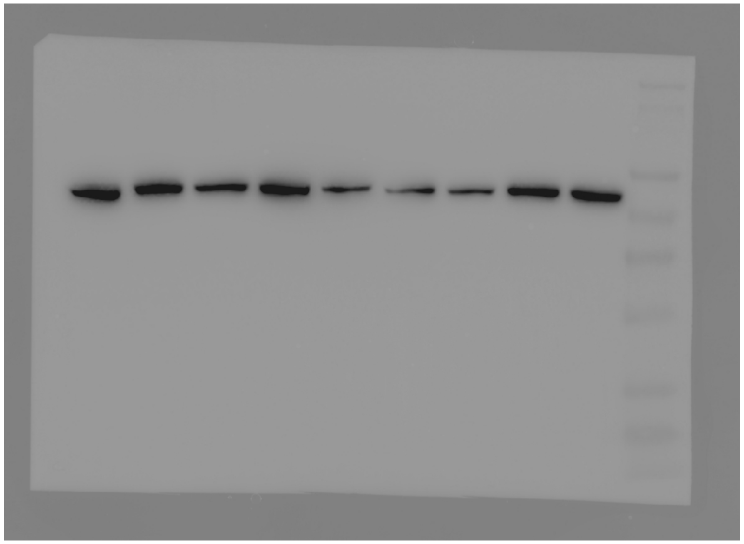

C3aR1 flox C3aR1 flox/ Ad-Cre

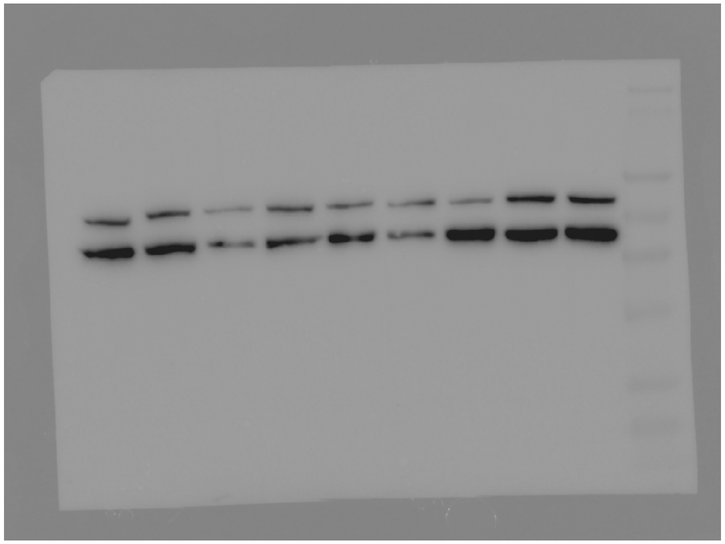

Supplement: Unedited blot and gel images [file jciinsight-9-178925-s007.pdf]
