# Supplementary material for: Adipsin and adipocyte-derived C3aR1 regulate thermogenic fat in a sex-dependent fashion
Source: JCI Insight. 2024 May 7;9(11):e178925. doi: 10.1172/jci.insight.178925 (PMC11382875; doi:10.1172/jci.insight.178925)
Supplement: Supplemental tables 1-2 [file jciinsight-9-178925-s008.pdf]

Supplemental Table 1. qPCR primer sequences

Gene	Forward	Reverse
Rps18	CATGCAGAACCCACGACAGTA	CCTCACGCAGCTTGTTGTCTA
C3ar1	TGACAGGTCAGCTCCTTCCT	CATTAGGAGGCTTTCCACCA
Ucp1	ACTGCCACACCTCCAGTCATT	CTTTGCCTCACTCAGGATTGG
Prdm16	CAGCACGGTGAAGCCATTC	GCGTGCATCCGCTTGTG
Ppargc1a	CCCTGCCATTGTTAAGACC	TGCTGCTGTTCCTGTTTTC
Ppargc1b	AGTCAGCGGCCTTGTGTCAA	ACTCTGGGACAGGGCAGCA
Adipsin	CGTACCATGACGGGGTAGTC	ATCCGGTAGGATGACACTCG
Alpl	CCAACTCTTTTGTGCCAGAGA	GGCTACATTGGTGTTGAGCTTTT
Cidea	TGCTCTTCTGTATCGCCCAGT	GCCGTGTTAAGGAATCTGCTG
Dio2	CAGTGTGGTGCACGTCTCCAATC	TGAACCAAAGTTGACCACCAG
Elovl3	TCCGCGTTCTCATGTAGGTCT	GGACCTGATGCAACCCTATGA
Serca1	TCATTGCCAACGCCATTGTG	CAGCCCGATAGACCTTTCCC
Serca2b	ACCTTTGCCGCTCATTTTCCAG	AGGCTGCACACACTCTTTACC
Ryr2	CTTCTGTGAGGACACCATCTTT	CCTCTCCTTCTCACTCTCTTCT
Sdhb	TAGCGGTCCTCAGGGTGAGA	CTGAAACTGCAGGCCGACTCT
Ckb	CTGTCTGGCAGGTACTACGC	TGCATGGAGATGACTCGCAG
Ckm	GAACCTCAAGGGTGGAGACG	GTTGAGAGCTTCCACGGACA
Ckmt1	TGACCCCTATTTTGGCTCCAG	TTGGGGATGCGGCTACAAAG
Ldhb	CAAAGGCTACACCAACTGGG	TTGAGGATGCACGGGAGACT
Pkm	CCTCCAGTCACTCCACAGAC	GCAATGATAGGAGCCCGAGG
Gpr3	ATCTACGCCTTTCGCAACCA	CGGGACCGGAATGGAATCTT
Fabp4	ACACCGAGATTTCCTTCAAACTG	CCATCTAGGGTTATGATGCTCTTCA
Dgat1	GGAGACCGCGAGTTCTACAG	CTCATGGAAGAAGGCTGAGG
Dgat2	TCTCAGCCCTCCAAGACATC	GCCAGCCAGGTGAAGTAGAG
Lpl	GGGAGTTTGGCTCCAGAGTTT	TGTGTCTTCAGGGGTCCTTAG
Gk	ACGGGCCATAAGTGTGTATTT	GAACGAAGTAGCAGCCATAAGA

Supplemental Table 2: C_T values in WT/Control Adipose Tissues at room temperature

**Approximate C_T for Gene**	**Subcutaneous Fat**	**Brown Fat**
Ucp1	23	15
Ckb	-	24
Alpl	-	26
Serca2b	23	21
Ryr2	31	29

C_T for RPS18: ~20
